# Systematic gastric surveillance in patients with familial adenomatous polyposis

**DOI:** 10.1016/j.vgie.2025.12.007

**Published:** 2025-12-26

**Authors:** Hicham Bouchiba, Dewkoemar Ramsoekh, Arthur S. Aelvoet, Barbara A.J. Bastiaansen, Evelien Dekker

**Affiliations:** 1 Department of Gastroenterology and Hepatology, Amsterdam UMC, Amsterdam, The Netherlands; 2 Cancer Center Amsterdam, Amsterdam, The Netherlands; 3 Amsterdam Gastroenterology Endocrinology Metabolism, Amsterdam, The Netherlands

## Abstract

**Background and Aims:**

Familial adenomatous polyposis (FAP) has shown a rising incidence of gastric cancer, particularly in the proximal stomach. We aimed to demonstrate a systematic protocol to improve gastric surveillance in FAP.

**Methods:**

The protocol consists of systematic gastric examination using white-light endoscopy and narrow-band imaging combined with patient repositioning from the left lateral to supine position in cases with carpeting fundic gland polyposis.

**Results:**

Three FAP cases illustrate the diagnostic value of this approach. In 2 cases, supine repositioning improved visualization of mucosa obscured by carpeting fundic gland polyps. In the third case, an ulcerated adenocarcinoma hidden among polyps with the patient in the left lateral position became visible only after repositioning.

**Conclusions:**

Systematic gastric surveillance with advanced imaging and patient repositioning can reveal lesions not visible on standard inspection. This approach may enhance early detection of premalignant and malignant gastric lesions in FAP.

## Introduction

**Figure 1:**
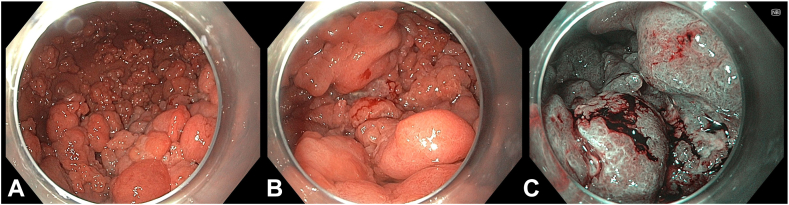
**A,** Carpeting fundic gland polyposis covering the proximal stomach, with gastric folds and normal gastric mucosa not visible. **B,** Whitish area of a low-grade dysplastic lesion over fundic gland polyps. **C,** The same lesion visualized with narrow-band imaging.

Source: Amsterdam UMC.

A concerning increase in gastric cancer diagnoses has been observed among patients with familial adenomatous polyposis (FAP) in Western countries over the past decade.^1^^,^^2^ Although gastric cancer in FAP has long been more prevalent in Asia, it was historically considered rare in the Western countries.^3^ Recent studies now report a rising incidence, with most cancers occurring in the proximal stomach, often in the setting of extensive carpeting fundic gland polyposis and associated with a poor prognosis.^1^^,^^2^ Detecting subtle dysplastic lesions within these areas is challenging. These lesions typically appear as whitish regions with a distinguishable mucosal surface pattern and are being identified more frequently, likely reflecting increased awareness of risk of gastric cancer, particularly proximal, in FAP (Fig. 1).^4^

**Figure 2:**
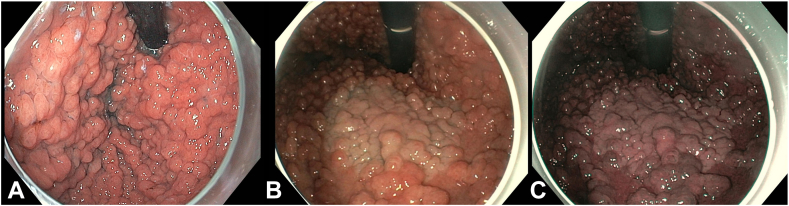
A 66-year-old male patient with familial adenomatous polyposis diagnosed with gastric cancer in the corpus. **A,** Bulky carpeting fundic gland polyposis in the corpus, with several whitish areas suspicious for dysplasia (left lateral position). **B and C,** In supine positioning, an ulcerative lesion suspicious for malignancy was identified; pathology confirmed adenocarcinoma.

Source: Amsterdam UMC.

To address the challenge of timely detecting relevant precursor lesions, we developed a systematic examination protocol (Video 1, available online at www.videogie.org). This includes gastric cleaning with an antifoam agent, administered before the procedure and reapplied through the working channel during the examination if visibility remains impaired. All examinations were performed with the patient under propofol sedation to improve patient comfort and to facilitate a stable and uninterrupted inspection, including during repositioning. After this, a detailed inspection is performed using both white-light endoscopy and narrow-band imaging (NBI) sequentially, preferably with a transparent cap attached to the gastroscope, combined with specific instructions to ensure complete mucosal coverage and repositioning from the left lateral to the supine position in patients with carpeting fundic gland polyposis for repeat detailed inspection (Fig. 2). Repositioning to supine may reveal dysplastic lesions among fundic gland polyps that were not visible in the initial left lateral position, as demonstrated in the video manuscript (Video 1).

## Case report

In the video manuscript (Video 1, available online at www.videogie.org), we present 3 FAP cases to demonstrate the utility of the systematic gastric examination protocol and the value of repositioning in detecting subtle gastric lesions.

The first case illustrates the complete stepwise inspection protocol. Starting in the antrum with the patient in the left lateral position, the stomach is examined antegrade along the greater curvature up to the fundus, followed by retroflexion to evaluate the fundus and cardia and continued along the lesser curvature back to the antrum. The same sequence is repeated using NBI (virtual chromoendoscopy) to improve mucosal visualization. This case demonstrates how potential dysplastic lesions may appear as whitish areas located among gastric folds or fundic gland polyps, which may otherwise be overlooked without a systematic approach.

The second case highlights the added diagnostic value of repositioning the patient to the supine position, particularly in patients with carpeting fundic gland polyposis. With the patient in the left lateral position, compressed gastric folds and overlying polyps prevented adequate inspection of a previously resected area. After repositioning the patient, gravity allowed the folds and polyps to fall away, fully exposing the scarred mucosa and enabling detailed evaluation of the prior resection site.

The third case involves a 66-year-old male patient with FAP with a surgical history of proctocolectomy and end-ileostomy. During routine endoscopic surveillance, 1 year after the previous endoscopy and before implementation of our current protocol, extensive carpeting fundic gland polyposis was observed, along with several whitish areas suspicious for dysplasia (Fig. 2). After repositioning the patient to the supine position, an ulcerative lesion suspicious for malignancy was detected among the fundic gland polyps along the greater curvature (Fig. 2). Biopsy samples confirmed adenocarcinoma, and a subsequent CT scan showed multiple liver metastases. The patient is currently receiving palliative chemotherapy.

Together, these 3 cases demonstrate that systematic inspection and supine repositioning can reveal dysplastic or malignant lesions that were not visible during standard left lateral examination.

## Conclusion

These cases highlight the importance of systematic and detailed gastric examination, with the use of advanced imaging and patient repositioning for the detection of premalignant and malignant gastric lesions in FAP. The overall effectiveness of this strategy in preventing gastric cancer in FAP remains uncertain and will require further evaluation. To our knowledge, no published data are available on the diagnostic yield of supine repositioning in FAP, and this approach is currently being evaluated within the International FAP Consortium.^5^

## Disclosure

The following authors disclosed financial relationships: B. A. J. Bastiaansen: Speaker for Olympus, Tillotts Pharma AG, and Ovesco Endoscopy AG. E. Dekker: Consultant for Olympus, Fujifilm, Ambu, InterVenn, Norgine, and Exact Sciences; speaker for Olympus, Norgine, IPSEN/Mayoly, Fujifilm, Steris, and Pentax; endoscopic equipment on loan from Fujifilm. All other authors disclosed no financial relationships.

## Supplementary data

Video 1 This video demonstrates a systematic gastric surveillance protocol for patients with familial adenomatous polyposis (FAP). The first part shows stepwise gastric examination, including white-light endoscopy and patient repositioning. The second part illustrates the effect of moving from the left-lateral to the supine position, improving visualization of the proximal stomach in cases with carpeting fundic gland polyposis. The third part presents a representative case of a 66-year-old male patient with FAP, in whom an ulcerated adenocarcinoma was detected only after supine repositioning, underscoring the clinical value of this approach.
